# Competition Between Emotional Faces in Visuospatial Working Memory

**DOI:** 10.1037/xlm0001330

**Published:** 2024-02-29

**Authors:** Marlene Poncet, Sara Spotorno, Margaret C. Jackson

**Affiliations:** 1 School of Psychology, University of Aberdeen; 2 School of Psychology and Neuroscience, University of St Andrews; 3 Psychology Department, Durham University

**Keywords:** face perception, visuospatial working memory, emotion, eye movements

## Abstract

Visuospatial working memory (VSWM) helps track the identity and location of people during social interactions. Previous work showed better VSWM when all faces at encoding displayed a happy compared to an angry expression, reflecting a prosocial preference for monitoring who was where. However, social environments are not typically uniform, and certain expressions may more strongly compete for and bias face monitoring according to valence and/or arousal properties. Here, we used heterogeneous encoding displays in which two faces shared one emotion and two shared another, and asked participants to relocate a central neutral probe face after a blank delay. When considering the emotion of the probed face independently of the co-occurring emotion at encoding, an overall happy benefit was replicated. However, accuracy was modulated by the nonprobed emotion, with a relocation benefit for angry over sad, happy over fearful, and sad over happy faces. These effects did not depend on encoding fixation time, stimulus arousal, perceptual similarity, or response bias. Thus, emotional competition for faces in VSWM is complex and appears to rely on more than simple arousal- or valence-biased mechanisms. We propose a “social value (SV)” account to better explain when and why certain emotions may be prioritized in VSWM.

Working memory (WM) plays an important role in our everyday life. It is a core component of multiple cognitive functions allowing us to retain, manipulate, update, and retrieve information according to the current situation (Baddeley, 2007). Signals evoking emotions are particularly relevant for updating information and adapting one’s behavior appropriately during social interactions. Visuospatial working memory (VSWM), in particular, plays a critical role in tracking and monitoring the identity and location of people to enable fluent scene and conversation processing, both when we play an active role and during passive observation. Facial expressions have been shown to influence WM for identity and location information, but much of what we know is based on task displays where all faces share the same emotion (Curby et al., 2019; Jackson et al., 2008, 2009, 2014; Spotorno et al., 2018). In the current study, we aimed to examine how faces with differing emotional expressions at encoding compete for VSWM resources to bias recall of who was where.

Several studies have investigated the role of facial expressions on how accurately the identity of faces is encoded and maintained in WM. Their results do not always align with each other and seem to depend on the specific methodology used (for reviews, see Gambarota & Sessa, 2019; Xu et al., 2021). In general, in such studies participants are asked to remember a set of faces and, after a short maintenance period, decide if a subsequently presented (probe) face corresponds to one of the memorized face identities or not. The emotional expression of the faces is irrelevant to the task but is shown to influence recall accuracy. Studies that have only asked for identity recall from visual WM have found that participants remember faces presented with angry expressions better than faces presented with happy or neutral expressions (Jackson et al., 2008, 2009, 2014; Thomas et al., 2014). In contrast, when participants are asked to recall both the identity and the location of a probe face (who was where; VSWM), relocation accuracy for faces presented with a happy expression was better than for faces presented with an angry expression (Spotorno et al., 2018; see also Curby et al., 2019).^1^

In the WM studies reviewed above, the stimuli were presented in an emotionally homogeneous context: during the encoding stage, participants saw either one stimulus at a time or multiple stimuli simultaneously, all evoking the same emotion. This is useful to determine emotion effects in the absence of competition between expressions with different arousal and valence properties. However, other work has shown that the emotional context in which a stimulus is encoded can affect how well it is maintained in memory. For example, Righi et al. (2015) asked participants to remember a happy or a fearful face embedded in a happy or a fearful scene. During the retrieval phase, participants were presented with a neutral face and they had to decide if its identity was shown during the encoding stage. They were also asked in which of three possible scenes the face was embedded. Performance was better for happy faces embedded in happy scenes, and the scene (happy or fearful) was better remembered when presented with a happy face. The authors interpreted this memory improvement to reflect a prosocial bias.

The effect of happy faces on memory performance for surrounding stimuli raises the question of how simultaneously presented faces with different emotional expressions compete for resources to influence recall accuracy in WM. It is well established that emotional stimuli in general, including faces, compete more strongly for resources and are cognitively prioritized over neutral stimuli (Compton, 2003; Vuilleumier, 2002, 2005). A few studies have directly investigated resource competition in WM between differently valenced faces by comparing WM performance for neutral versus emotional faces shown together at encoding. Thomas et al. (2014) presented a single angry or happy face among three neutral faces, in a visual identity task with no spatial component. Their results showed that angry singletons were remembered better than happy singletons, replicating the angry advantage in visual WM for face identity (Jackson et al., 2008, 2009, 2014). However, WM accuracy for neutral faces was similar whether they were encoded alongside an emotional (angry or happy) singleton, or within a homogeneous display of neutral faces. That is, the authors did not find any evidence of competition in WM. If disproportionately more resources were allocated to the emotional singleton than to the neutral faces due to its strong valence, then WM for neutral faces encoded with an emotional singleton should have been worse than if the neutral faces were encoded with other neutral faces. On the other hand, Lee and Cho (2019) found that when two neutral and two emotional faces (fearful or happy) were presented simultaneously, WM performance improved for the emotional faces and decreased for the neutral faces compared to a homogeneous display in which all faces expressed the same emotion. This suggests that attentional resources in WM can be preferentially allocated to emotional over neutral faces. Interestingly, this “memory trade-off” disappeared when the faces were presented sequentially, that is, when there was less direct competition during the encoding period.

Results from a VSWM study in which both identity and location were task relevant also show some evidence that emotional information is prioritized over neutral information (Terburg et al., 2012). In this experiment, participants had to remember the identity and location of four angry or four happy faces and their four neutral counterparts that were presented with them. The faces briefly disappeared and then all reappeared at the top of the screen for participants to relocate them to their previous location in any order they chose. The focus of the study was on the role of anxiety-driven biases toward or away from the emotional faces, and direct statistical comparisons were not provided between emotional versus neutral faces encoded in the same display. However, numerically, relocation accuracy was better for happy (64.8%) than for angry (60%) faces, consistently with the happy advantage found using homogeneous displays (Spotorno et al., 2018). VSWM for neutral faces was also slightly poorer when they were encoded in the presence of angry faces (63.5%) compared to happy faces (65.6%). Lack of direct comparisons between angry and happy faces (not just with neutral), however, means that there is still insufficient understanding of emotion competition effects in WM. Our current study addresses this by examining for the first time how expressive faces with different emotions compete for VSWM resources. This will also allow us to tease apart different theoretical accounts of such competition.

Most WM studies using emotional faces have interpreted their results according to whether face valence was positive or negative (or neutral). It has also been proposed that stimulus arousal (intensity) influences WM performance. A good example of this is observed in episodic memory where past events are remembered more often and with increased vividness if they were highly arousing (LaBar & Cabeza, 2006). While both arousal and valence information contribute to enhancing memory, they rely on distinct neural processes (Kensinger & Corkin, 2004). Arousal information seems supported by the amygdala network, whereas valence seems supported by the prefrontal network, although it must be noted that the way in which the amygdala is activated by arousing stimuli also depends on the valence of the stimuli (Mickley Steinmetz et al., 2010). Mather and Sutherland (2011) developed the arousal-biased competition (ABC) account, which proposes that arousal, elicited internally or externally, amplifies competition between low- and high-priority stimuli. The priority of a stimulus, as defined by Mather and Sutherland, is the combination of perceptual features (visual saliency) and top-down features such as its task relevance, stimulus’ expectancy, or its emotional and social relevance. Depending on these factors, the processing of a stimulus is either enhanced (high priority) or weakened (low priority). According to the ABC account, arousal further increases this priority bias.

In our study, we presented only emotional faces and all four faces were task relevant, so there was no strong priority bias compared to a display where both emotional and neutral faces are presented or in which only some faces are task relevant. We used angry, happy, sad, and fearful faces and paired these directly with one another in all possible combinations. Neutral faces were not shown at encoding but were used to probe VSWM at retrieval. Based on the evidence of a prosocial, happy face advantage in VSWM in emotionally homogenous displays (Spotorno et al., 2018; Terburg et al., 2012), we expected happy faces to be prioritized over all concurrently presented negative (angry, fear, and sad) faces. According to the ABC account, this effect should be stronger when happy faces are paired with low-arousing (sad) faces. Moreover, when all faces are negatively valenced, the role of arousal may be observed more clearly, and we expected to see a VSWM advantage for high-arousal faces (e.g., fear) over lower-arousal faces (e.g., sadness).

VSWM performance was assessed using the same paradigm as in Spotorno et al. (2018). We measured face relocation accuracy using a categorical measure (whether the face was relocated within the correct area or not) and a continuous measure (how close was it relocated to the correct location). We also recorded participants’ eye movements to examine whether there was any systematic oculomotor behavior dependant on the emotion carried by the faces during the encoding period. More specifically, recording eye movements should help us to explore the potential role of any fixation biases during the encoding period in any emotional memory effects found. It has previously been shown that participants fixate emotional pictures first and longer than neutral pictures (Calvo & Lang, 2005; Nummenmaa et al., 2006; Rösler et al., 2005; Thomas et al., 2014). For instance, in Thomas et al.’s (2014) study where an angry or happy face singleton was encoded alongside three neutral faces, proportionately more fixations on the angry singleton than on the neutral faces resulted in poorer visual WM for neutral faces. However, previous eye-movements results are not clear when positive and negative pictures are presented together (Astudillo et al., 2018; Fernández-Martín & Calvo, 2016).

Finally, to confirm that our results were the consequence of a difference in VSWM and were not due to a potential low-level perceptual or response bias, we conducted a second experiment using a task that did not contain any memory component. Participants were asked to match the identity of a neutral face to one of two concurrently presented emotional faces used in the first experiment. If the same pattern of results is found across both experiments, this would suggest that responses were biased by one type of emotion regardless of the task (VSWM in Experiment 1 and perceptual identity matching task in Experiment 2). Otherwise, it would confirm that the effects found in the main experiment (Experiment 1) reflect the competition for resources in VSWM.

## Experiment 1: VSWM Task

### Method

#### Participants

Thirty-six volunteers (24 women; age: *M* ± *SD* = 21 ± 2 years) participated in this experiment. Two additional participants were rejected, one because they did not complete the experiment, and one because their eye movements were not recorded. The experiment received the approval of the ethical committee of the School of Psychology, University of Aberdeen. All participants had normal or corrected to normal visual acuity and provided written informed consent. They were reimbursed £5 for their participation.

Even though it can be argued that some participants show poor performance, we did not reject any participant based on this criterion. The main reason is that in this experiment, accuracy depends on the size of the area that we consider as being a correct relocation (see Data Analysis section) so there is no chance level per se. Instead, each participant’s accuracy changes depending on the size of the correct area that we choose. In addition, it is difficult to set a threshold between “good” and “bad” performers as there is not a strict division but a continuum in accuracy performance among our group of participants.

#### Material

The setup of the experiment was similar to Spotorno et al. (2018). Participants were seated in a dimly lit room with their head resting on a chinrest 40 cm away from a touchscreen computer (EliteOne 800, 1,920 × 1,080, screen width = 51 cm). Their eye movements were recorded using a tower-mounted EyeLink 1000 Plus (SR Research) sampling at 1,000 Hz. Although participants viewed the stimuli with both eyes, only their dominant eye was recorded (the left eye for 11 participants). The face stimuli (2.4 × 3.4 cm, corresponding to 3.4° × 4.9° of visual angle) were presented on a white background using Experiment Builder (SR Research). The locations of the faces were randomly generated before the experiment, but there was always a minimum of 12° of visual angle between the center of two faces and a central area of 7° of visual angle around the fixation cleared from any stimulus (Figure 2). The identity, emotional expression, and location of the faces were counterbalanced: 12 lists, generated in Spotorno et al. (2018), were reused in this experiment.

We used a set of 40 grayscale face stimuli composed of eight Caucasian male individuals expressing five different emotions: neutral, happy, sad, fear, and angry. The identities of the faces were the same as the ones in Spotorno et al. (2018). The faces were taken from the Radboud Faces Database (Langner et al., 2010), cropped into an oval shape (no hair was visible), and set to grayscale. The mean luminance and contrast were equalized across all faces such that low-level properties cannot be responsible for the potential effects observed in the results.

**Figure 1 fig1:**
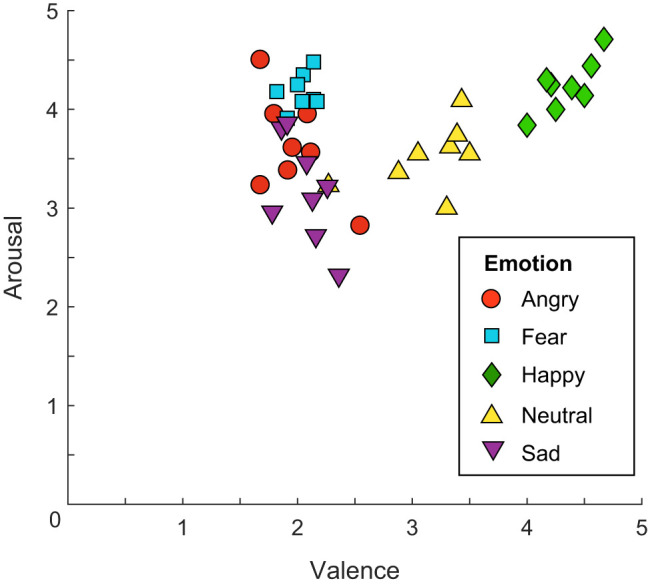
Arousal and Valence Ratings of the Faces Used in the Study *Note*. The ratings, made on a 5-point scale, are taken from “Presentation and Validation of the Radboud Faces Database,” by O. Langner, R. Dotsch, G. Bijlstra, D. H. J. Wigboldus, S. T. Hawk, and A. van Knippenberg, 2010, *Cognition & Emotion, 24*(8), pp. 1377–1388 (https://doi.org/10.1080/02699930903485076). Copyright 2010 by Taylor & Francis Online. Here we show the ratings for the eight face identities used in this study, across the four emotional expressions, shown at encoding, plus the neutral expression, shown at retrieval. See the online article for the color version of this figure.

The face arousal (intensity) and valence ratings provided by the Radboud Faces Database are illustrated in Figure 1. In the Discussion section, we comment on our results according to the average arousal (happy: *M*_Int_ = 4.24; angry: *M*_Int_ = 3.63; fear: *M*_Int_ = 4.18; sad: *M*_Int_ = 3.18) and valence (happy: *M*_Val_ = 4.34, sad: *M*_Val_ = 2.07, fear: *M*_Val_ = 2.03, angry: *M*_Val_ = 1.98) of each emotion. Using an average might not reflect what is happening in an individual trial, since faces with the same emotion do not always convey the same degree of arousal or valence, as this may vary depending on the specific image. However, these ratings are consistent across the different face identities so if there is a systematic bias in performance due to the arousal or valence carried by an emotion, this should be visible in the average scores per emotion regardless of the identity of the faces.[Fig fig1]

#### Procedure and Design

**Figure 2 fig2:**
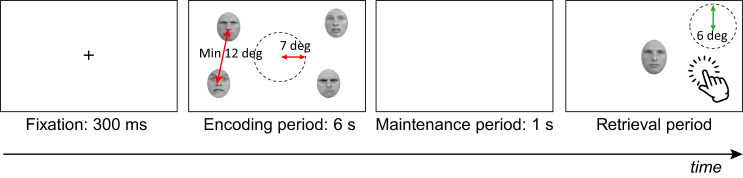
Paradigm of the Experiment *Note*. Participants had to fixate the center of the screen for 300 ms before four emotional faces were presented on the screen (encoding period). The faces (from four identities and two emotions) were separated by a minimal distance of 12° of visual angle and an area of 7° of visual angle was left empty in the center of the screen. After a blank interval of 1 s (maintenance period), a neutral probe face with the same identity as one of the four faces presented during the encoding period appeared in the center of the screen. Participants had to relocate where the probe face was during the encoding period using the touchscreen (retrieval period). A response was considered correct if it was relocated within 6° of visual angle from the center of the original location of the face (illustration not drawn at scale). Emotional faces were taken from the Radboud Faces Database published in “Presentation and Validation of the Radboud Faces Database,” by O. Langner, R. Dotsch, G. Bijlstra, D. H. J. Wigboldus, S. T. Hawk, and A. van Knippenberg, 2010, *Cognition & Emotion, 24*(8), pp. 1377-1388 (https://www.tandfonline.com/doi/abs/10.1080/02699930903485076). Copyright 2010 by Taylor & Francis. See the online article for the color version of this figure.

At the beginning of the experiment, the eye tracker was calibrated for each participant using a nine-point procedure, which was then validated. Each trial started with a fixation cross presented in the center of the screen (Figure 2). Participants had to maintain the fixation cross for 300 ms within 1° of visual angle for the stimuli to be presented. Failing to do so would trigger a new eye calibration. Participants were shown four to-be-remembered faces of different identities at random screen locations for 6 s. To avoid any effect due to one single emotion being more distinctive and better remembered (Quinlan et al., 2017), two faces carried one emotion while two other faces carried a different emotion (six paired combinations of angry, fear, happy, and sad). After a blank (white screen) maintenance interval of 1 s, one of the four faces was presented at the center of the screen (a location never occupied at encoding) with a neutral expression (probe face). Participants had to relocate this probe face using the touchscreen to where it was first presented, matching the correct identity with the correct location. They were asked to be as precise as possible and could relocate the face as many times as they wanted before pressing the space bar to confirm their response and start a new trial. It is important to note that emotion was not relevant for the task, as participants were asked to remember the bound identity and location of the four faces (who was where), not their expression. In all trials the probe face always shared identity with one of the faces shown at encoding.[Fig fig2]

There was a total of 12 conditions in the experiment (Six Emotional Pairings × Two Tested Emotion in Each Pairing). Each pairing condition was presented 32 times in a random order. The identity of the probe face was chosen equally from one of the four faces to-be-remembered. This way, the number of probe faces presented with one or the other emotion at encoding was equal in each pairing condition. For example, there were 32 trials with two happy and two angry faces to memorize and participants were tested on 16 neutral faces matching the happy identity and 16 neutral faces matching the angry identity. Out of these 16 neutral faces, each face identity was presented twice. Participants were trained on 12 trials and tested on a total of 192 experimental trials.

Participants were also asked to respond to three questionnaires as a standard procedure in the lab. The first questionnaire, the Positive and Negative Affect Schedule (PANAS; Watson et al., 1988) was given prior to the main experiment to measure mood at the time. The second questionnaire, the autism-spectrum quotient (AQ; Baron-Cohen et al., 2001) and the third, the Liebowitz Social Anxiety Scale (LSAS; Mennin et al., 2002) were given after the memory experiment. Participants were informed that they could skip a question if they did not wish to answer it. All questionnaires were completed on the computer. Although we collected this information, we did not test any hypothesis in relation to the study presented here and so we do not report the results of the questionnaires.

#### Data Analysis

##### Behavioral Performance

We analyzed two measures of VSWM performance: accuracy and precision (Spotorno et al., 2018). A response was considered correct if the probe face was relocated within a predetermined “safe zone.” This region was the original face surrounding area in which no other face was presented. Here, 12° of visual angle separated two faces from center to center, so a face was considered correctly relocated if its center was within 6° of visual angle from its original position (Figure 2). When correctly relocated, we also measured the distance between the original and relocated location (center to center) to obtain a measure of the precision of the response within that region. We performed two types of analyses:

1 *Overall effect of face expression on VSWM capacity*. This analysis tested whether VSWM performance was affected by the emotion that the neutral probe face showed at encoding, irrespective of the emotion of the other faces in the display.
2 *Competition effects*. Here we examined whether and how emotions compete for resources in VSWM. In this analysis, we were interested in determining if there was any performance unbalance in a given emotional pairing between the relocation of one or the other emotional face. To this end, we calculated the difference in relocation performance for the neutral face that was presented with one emotion versus the other emotion in the paired display at encoding. For example, when angry and fear faces were presented, we calculated VSWM performance when the neutral face was encoded as angry versus when it was encoded as fearful. We then computed the difference between these two scores and compared it to 0 (which would indicate no bias in VSWM).

Statistical analysis was performed using SPSS. Because the data was skewed and therefore not normal, we used the Friedman test as a nonparametric equivalent of a repeated-measures analysis of variance (ANOVA), to test the role of face emotion on VSWM performance.^2^ If significant, subsequent nonparametric Sign tests with Bonferroni correction for multiple comparisons were performed (all results are reported after correction by multiplying the *p* values by six, which corresponds to the number of comparisons performed). We also report Kendall’s coefficient of concordance (Kendall’s *W*), which can be used as an effect size of the Friedman test (Kendall’s *W* ranges from 0 to 1, with higher values indicating a stronger agreement among rankings). To test for a difference in performance between emotions in the biased competition analysis, we used one sample Sign tests (with a comparison to 0 for no difference). These results are also reported after Bonferroni correction by multiplying the *p* values by six.

##### Eye Movements

To test for a potential emotional bias during encoding, we analyzed participants’ eye-movements during the encoding period. A fixation was considered on a face stimulus if it was on or 1° of visual angle away from the oval-shaped stimulus image. Only trials in which the total fixation time was at least 2 s (out of the 6 s encoding period) were included. We computed three measures of eye-movements.

First, to determine whether participants looked longer at one or the other emotion during the encoding period, we calculated the total fixation time spent on each face image. We then pooled the fixation time for the faces that shared the same emotion and calculated the percentage of time spent on the two emotions (equal time should be 50%). Second, we tested whether there was any bias in fixating one emotion preferentially at the beginning of the trial which could reflect some saliency or attentional effect in the face stimuli. For this, we computed for each pair of emotions the percentage of trials in which the participants first fixated on one or the other emotion in the display. If they randomly fixated one emotional face for each trial, we should observe no bias (50%). This would suggest that the stimuli have a similar saliency, and that no emotion is prioritized when the faces appear. Finally, to test for a potential recency effect that might explain some of our results, we determined which emotion was last fixated on in each emotional pairing and computed a percentage across all trials and participants.

#### Transparency and Openness

All experimental programs, stimuli, data, and analysis code are publicly available on the Open Science Framework (OSF) at https://osf.io/gr96x/ (Poncet & Jackson, 2023). Results were analyzed using MATLAB (The MathWorks Inc.). All figures were created using the Gramm plotting toolbox (Morel, 2018). The design of the study and its analysis were not preregistered.

### Results

#### Overall Effect of Face Expression on VSWM Performance

We first tested whether the emotion of a face at encoding (angry, fear, happy, or sad) influenced VSWM overall regardless of the other emotion copresent at encoding. If the prosocial advantage found in displays with homogeneous facial expressions (Spotorno et al., 2018; Terburg et al., 2012) is also present in displays with heterogeneous emotional faces, we should replicate a VSWM benefit for happy faces.

**Figure 3 fig3:**
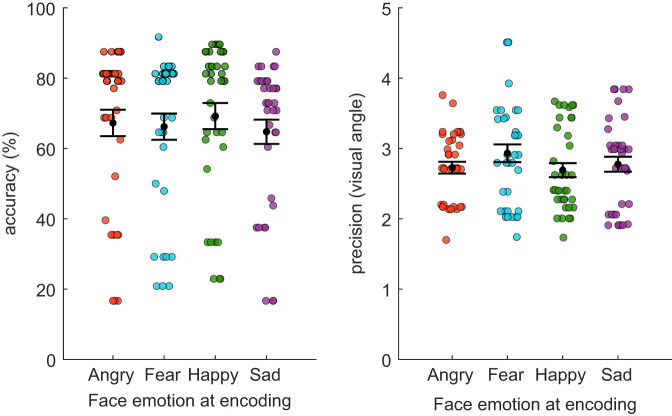
Overall VSWM Performance in Experiment 1 *Note*. Mean of correct relocations (left) and precision of the relocations (right) of the neutral probe face depending on the face expression at encoding, independently of the co-occurring emotion at encoding. Each circle represents one participant’s performance. The *M* and *SEM* are represented in black. Note that there is no defined “chance-level” in this experiment (see text for details). VSWM = visuospatial working memory; *SEM* = standard error of the mean. See the online article for the color version of this figure.

*Accuracy*: Participants were on average 67% correct in matching the identity of the probe face to its original location (Figure 3). There was a large disparity in performance: some participants were very good at the task, with up to 85% correct responses while the lowest performance was 19%. The results of the Friedman test showed a main effect of emotion on VSWM performance (Friedman’s *Q* [3, *N* = 36] = 14.71, *p* = .002, Kendall’s *W* = 0.14). Faces encoded with a happy expression were better relocated than faces encoded with a fear (*Z* = 3.10, *p* = .011) or sad (*Z* = 2.83, *p* = .028) expression. Moreover, sad faces were less well relocated than fear faces (*Z* = 2.65, *p* = .048). There was no other difference in performance between the other pairs of emotional expression (all *p*s > .90).[Fig fig3]

*Precision*: The relocation of the probe face within the correct face region was on average 2.89° of visual angle away from the original location (Figure 3), which is around half the distance accepted for a correct answer (6°). There was no effect of emotion on relocation precision (Friedman’s *Q* [3, *N* = 36] = 4.17, *p* = .25, Kendall’s *W* = 0.04).

*Swap errors*: To determine the type of errors that participants made, we looked at whether some of the incorrect relocations were within another face area. This corresponds to a misbinding or swap error (Pertzov et al., 2012; Spotorno et al., 2018): participants remembered a location but reported it with a wrong face identity. This analysis revealed that 82% of errors were swap errors, and these were not distributed similarly across the four emotions (Friedman’s *Q* [3, *N* = 36] = 7.79, *p* = .048, Kendall’s *W* = 0.07). Participants showed a tendency for more swap errors for sad (84%) than happy (81%) or angry (80%) faces, but these effects did not survive multiple comparisons (both *p*s > .13).

##### Summary

Participants were overall better at relocating a face that was previously seen with a happy than a sad or fearful expression, and better at relocating a face encoded with a fearful than a sad expression. When they did not relocate the probe face accurately, participants usually exchanged a face location with another one (misbinding or swap error between face identity and location). Notably, the advantage for relocating happy faces and disadvantage for relocating sad faces matches both the pattern of valence and arousal scores with happy faces having the highest scores and sad faces the lowest (note that we are not considering direct competition effects here, rather more inherent stimulus properties). However, valence and arousal ratings do not fully explain the pattern of performance for angry and fearful faces. While these two facial expressions are both rated low in valence (i.e., negatively), fearful faces are rated higher in arousal than angry faces (Langner et al., 2010). If VSWM is better with higher arousal, we would expect better performance for fearful faces compared to angry faces. However, we found the opposite results. Thus, in general, the happy benefit found here indicates that prosocial signals may be a stronger driver than just arousal for remembering who was where.

#### Competition Effects

VSWM performance depending on the emotion of the test face at encoding and the co-occurring emotion is presented in Table 1. To determine whether and how the emotions carried by faces compete for resources in VSWM, we calculated the difference in performance between the two emotions presented simultaneously. No difference in performance would indicate that there is no bias between the two emotions and the stimuli are equally prioritized in VSWM. Alternatively, a difference in performance would indicate that VSWM is biased for one emotion over the other, indicating emotion-specific prioritization of resources.[Table tbl1]

##### Behavioral Results

**Figure 4 fig4:**
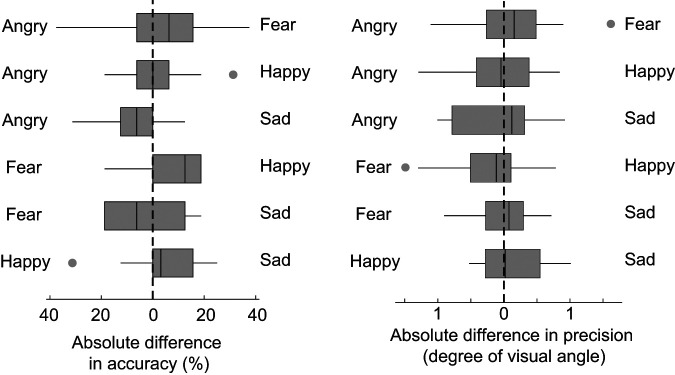
Performance Bias Between Pair of Emotions in Experiment 1 *Note*. Absolute difference in relocation accuracy (left) and precision (right) according to the emotion pairings presented at encoding (each boxplot represents one condition). The direction of the effect corresponds to the emotion indicated on the side of the boxplot. For example, in the happy–sad condition, participants were more accurate to relocate the neutral face that was presented with a sad than with a happy expression.

*Accuracy*: We first compared VSWM performance for the conditions that include happy faces to test for a potential prosocial bias (which would favor happy faces over all other negative emotions), accompanied or not by an arousal bias (which would favor happy faces when paired with any other emotion, fearful faces when paired with angry or sad faces, and angry faces when paired with sad faces). We observed better VWM performance for happy faces in the happy–fear condition (*Z* = 3.65, *p* = .001), showing a prosocial benefit despite similar arousal levels for these two emotions (Figure 4a). However, when happy faces were presented with sad faces, participants relocated the neutral probe face around 6% less accurately when it matched the identity of a happy face compared to a sad face (*Z* = 2.68, *p* = .044). The opposite results would have been expected from a prosocial or an arousal account. Furthermore, while either account would predict an advantage of happy versus angry faces, there was no bias in the happy–angry condition (*Z* = 0.46, *p* = 1)*.*[Fig fig4]

We then compared VSWM performance when two negative emotions are paired and thus only considerably differ by their degree of arousal. This allows us to better isolate the role of arousal in the allocation of resources in VSWM. In the angry–sad condition (Figure 4a), participants relocated the neutral probe face 8% more accurately when it matched the identity of an angry face compared to a sad face (*Z* = 3.62, *p* = .002); this aligns with higher arousal ratings for angry than sad faces. However, no evidence for biases was found for the remaining angry–fear and sad–fear pairings (all *p*s > .31); this is coherent with a valence account, whereas an arousal account would instead predict an advantage for fearful faces in both cases.

*Precision*: There was no evidence for biases in precision performance between any of the emotional pairs tested (Figure 4b; all *p*s > .21). This suggests that emotions did not directly compete for resources in maintaining precise identity-location information.

##### Eye Movements

It is possible that participants might be looking longer at one emotion compared to the other one during the encoding period, such that some emotional faces would be encoded for longer—and thus better—than others, as information accumulates across fixations (e.g., Tatler et al., 2005). This could be due to a bias in allocation of overt attention (engagement) at encoding (e.g., Posner, 1980) reflecting emotion prioritization, or to difficulties in disengaging attention from some emotions, as Becker et al. (2019) found for angry faces. These effects might be at the origin of the competition results that we report. We thus analyzed participants’ fixation time on each pair of emotional faces during the encoding period.

**Figure 5 fig5:**
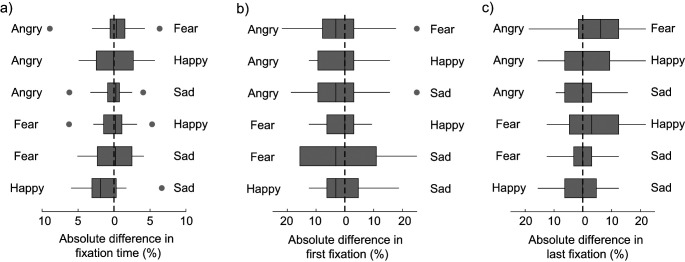
Eye-Movements Bias Between Pair of Emotions in Experiment 1 *Note*. Difference in (a) total fixation time, (b) first emotion fixated on, and (c) last emotion fixated on, according to the emotion pairings presented at encoding. There was no bias except in the happy–sad pairings where happy faces were fixated longer than sad faces.

This analysis reveals that in each of the six pair conditions, participants spent the same amount of time (50%) fixating on the two pairs of emotional faces (Figure 5a; all *p*s > .99), except when happy and sad faces were presented together. In that condition, participants fixated on happy faces longer than on sad faces (51% vs. 49%, *Z* = 3.35, *p* = .005; Figure 4c). Although consistent across participants, this effect is small and unlikely to be at the origin of the performance bias in happy–sad displays. Indeed, we found better performance for sad than for happy faces in that condition. Moreover, the other differences observed in VSWM performance cannot be explained by fixation time either. Therefore, differences in VSWM accuracy cannot be attributed to a difference in attentional bias either due to overt engagement or disengagement during the encoding period.[Fig fig5]

In addition, we tested whether in each pair of emotions one emotional expression was consistently first fixated on. The results showed no such bias (Figure 5b; all *p*s > .49), confirming the absence of a saliency or an attentional bias in the stimuli. We also tested whether our results might be explained by a recency effect by analyzing which emotion was last fixated in the encoding display. Again, we did not find any bias (Figure 5c; all *p*s > .11) which could have explained differences in VSWM performance.

##### Summary

Our results show that VSWM performance is not equal across pairs of emotion and that differences in performance are not due to differences in fixation time during the encoding period. This supports the idea that competition in VSWM depends on the emotion of the stimulus even when emotion is not relevant for the task. However, our results cannot simply be explained by differences in either valence or arousal between emotions.

One potential confound that we need to consider is whether our results reflect a difficulty in matching the identity of the neutral probe face with that of the emotional face at encoding. Indeed, applying an index which quantifies the structural similarity between images (Wang et al., 2004) to the face database from Yin et al. (2006), Chen et al. (2011) showed that neutral faces were structurally more similar to their sad and angry counterparts than to their fearful counterparts, while neutral and happy face were structurally the least similar. Thus, the degree of perceptual similarity between the neutral face in our experiment and its expressive counterpart during the encoding could affect our results. Another potential confound is whether any emotion effects in VSWM could be explained by a response bias in reporting one emotion over another. If participants are more likely to relocate the neutral face to the location of a certain emotion (regardless of whether it matches the identity of the face), VSWM performance will be higher for that emotion. That is, the bias in performance might be due to a difference in response bias (reporting preferentially one emotion over another), not a VSWM bias. We tested these two possibilities in a second experiment using the same faces as in Experiment 1, but this time no memory component was involved in the task.

## Experiment 2: Perceptual Identity Matching Task

Participants were asked to match the identity of a neutral face with one of two concurrently presented emotional faces that carried two different expressions as per the six expression pairs used in Experiment 1. The identity of the neutral face always matched one of the two expressive faces. If, as mentioned, the overall VSWM advantage for happy faces and disadvantage for sad faces can be explained by happy faces being easier to match with a neutral face and sad faces being more difficult, then accuracy and reaction times (RTs) would be expected to be better/faster when the neutral face matches the happy face and worse/slower when the neutral face matches the sad face. We can also measure performance for each pairing conditions to assess the presence of any perceptual (matching difficulty) or response bias (reporting preferentially one emotion over another) between two emotional expressions. If these factors can explain the competition effects between pairs of emotions found in Experiment 1, we would expect to replicate better matching performance (and/or higher response bias) for angry faces in angry–sad pairs, for sad faces in sad–happy pairs and for happy faces in happy–fear pairs.

### Method

#### Participants

Thirty-five volunteers (29 women; age: *M* ± *SD* = 19 ± 2 years) participated in this experiment. Two participants were rejected from the analysis due to very poor performance (*d*′ around 0). The experiment received the approval of the ethical committee of the School of Psychology, University of Aberdeen. All participants had normal or corrected to normal visual acuity and provided written informed consent. They were undergraduate Psychology students who completed the experiment during a group-based lab session.

#### Procedure and Design

Participants were presented with two grayscale emotional faces, one on each side of a fixation cross that was placed in the center of a white background screen. The two faces always carried different identities and expressions. We used the same faces as in Experiment 1, with the same six possible emotion pairs (happy–angry, happy–sad, happy–fear, angry–sad, angry–fear, and fear–sad). After 2 s preview of the emotional face pair, a neutral face appeared below the fixation cross and the face pair. All faces had the same size on the screen (2 × 3 cm) and participants were sitting approximately 40 cm away from the screen (there was no chinrest). The two emotional faces were presented in the middle of the screen, each 1.8 cm away from the center of the screen (so they were separated by 3.6 cm, center to center). The neutral face was presented 4.8 cm below the center of the screen. Participants had to determine as quickly and accurately as possible which of the emotional faces matched the identity of the neutral face. Importantly, the two emotional faces and the neutral face remained present together on the screen, so the task did not involve WM but relied on perceptual matching of face identities. The participants responded by pressing one of two designated keys corresponding to the left- or right-located face. The faces remained on the screen until a response was made. There was no feedback, and each trial was self-initiated. Participants were trained on 12 trials and tested on a total of 240 trials (40 trials per emotion pair, presented in a random order). In each emotion pair, the neutral face matched the identity of each emotional face an equal number of times (i.e., within the 40 happy–sad trials, the neutral face matched the happy face identity on 20 trials and the sad face identity on 20 trials, randomized). Whether the matching face was on the left or right of the face pair was also fully counterbalanced. There were no nonmatch trials.

#### Data Analysis

We analyzed the data by first considering the general effect of emotional expression on performance regardless of the other co-occurring expression to test whether any emotional expression was easier to match with the neutral face. Trials were classified depending on which emotion was carried by the face that matched the identity of the neutral face. For these four categories of trials (angry, fear, happy, and sad), we computed accuracy (percent correct) and RTs for correct responses. We also computed a balanced integration score (BIS; Liesefeld & Janczyk, 2019) that controls for speed–accuracy trade-offs by combining these two measures (accuracy and reaction time). As in Experiment 1, we used Friedman tests to assess the role of face emotion on overall performance, followed by sign-rank tests when relevant.

In a second analysis, we examined competition effects between the two emotional faces for each of the six possible emotion pairs. Unlike in Experiment 1, because there were only two possible responses (one correct and one incorrect), we were able to compute a sensitivity index (*d*′) and a decision criterion (response bias) for each emotion pair (Green & Swets, 1966). *d*′ was calculated as the difference in *z*-scores between hits and false alarms for each pairing condition separately. For example, in a pairing with an angry and a happy face, a correct match between the neutral and the angry face was considered a hit and an incorrect match (i.e., choosing the happy face) was considered a false alarm. A *d*′ of 0 means that the number of hits was the same as the number of false alarms. Higher *d*′ indicates better discriminability performance. Response bias was also calculated for each pairing condition separately as:

$$\mathrm{Response}\;\;\mathrm{criterion}=\;-0.5\times (z_{\mathrm{Hits}}+z_{\mathrm{False}\;\mathrm{alarms}}),$$ 1

with the same definition for hits and false alarms as used to compute *d*′. A response criterion of 0 indicates no response bias; the more it deviates from 0, the higher the responses are biased. Applied to the perceptual identity-matching task, for example in the angry–happy pairing, no bias would indicate that participants are equally likely to choose the angry or the happy face. A bias different from zero, toward the happy face, would indicate that participants match preferentially the neutral face with the happy face (i.e., more often than with the angry face), regardless of the type of trial (correct or incorrect).

To test whether the neutral face is matched with one emotional expression faster than another, we also calculated the difference in RT between each paired emotional expressions for all correct responses. A difference in RT would suggest that one emotional expression is perceptually more similar to the neutral face than the other. To test this, we applied Friedman tests on *d*′ performance, followed by sign-rank tests when relevant. To specifically assess the presence of bias within each pairing condition, the measures of response bias and differences in RT were compared to 0 (no difference between the two emotions) using one-sample sign-tests for each pairing condition separately. All tests are reported after Bonferroni correction for multiple comparisons by multiplying *p* values accordingly.

### Results

#### Overall Matching Performance

To assess whether one facial emotion was easier to perceptually match with its neutral counterpart, in this analysis we focus on the effect of the emotion carried by the matching face stimulus regardless of the other emotion it was presented with (i.e., collapsed across pairing conditions). Participants’ overall accuracy in matching the identity of the neutral face with its corresponding emotional face was around 90%. Accuracy performance was not equal across all emotions (Table 2; Friedman’s *Q* [3, *N* = 33] = 9.70, *p* = .02, Kendall’s *W* = 0.10) but post hoc comparisons did not reach significance (all *p*s > .07). The analysis of RT showed that the emotion carried by the matching face did not affect the speed at which participants matched the neutral face with the correct emotional face identity (Table 2; Friedman’s *Q* [3, *N* = 33] = 4.40, *p* = .22, Kendall’s *W* = 0.04). To examine speed-accuracy trade-offs, we also computed the BIS (Table 2). BIS measures the relative performance difference between conditions. A BIS of 0 corresponds to equal performance across conditions, a high BIS means that performance in that condition is better than in the other conditions, and a negative BIS means that performance in that condition is lower than in the other conditions. Statistical analysis suggested no effect of the matching face emotion in discrimination performance (Friedman’s *Q* [3, *N* = 33] = 5.98, *p* = .11, Kendall’s *W* = 0.06).[Table tbl2]

#### Competition Effects in Matching Performance

**Figure 6 fig6:**
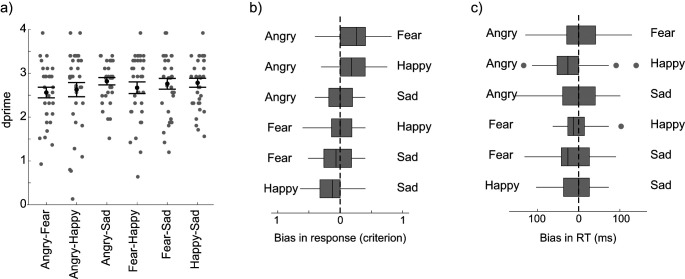
Performance Bias Between Pair of Emotions in Experiment 2 *Note*. Mean discriminability (*d*′) performance (a) in the identity matching task (Experiment 2). Each gray circle represents one participant’s performance. The *M* and *SEM* in each pairing condition are indicated in black. Response bias (b) and RT difference (c) between each pair of emotions. The two emotions present in the display are indicated on each side of the boxplot with the vertical dashed line indicating no bias. The closer the boxplot is to the corresponding emotion label, the larger the response is biased toward this emotion and away from the other emotion in the display (e.g., in Part b, participants’ responses are biased toward happy faces in a happy–sad pair). *SEM* = standard error of the mean.

In this analysis, we compare performance in the perceptual identity matching task separately for the six emotional pairing conditions. Discriminability performance (*d*′) was around 2.7 for all pairs of emotions (Figure 6a, Friedman’s *Q* [5, *N* = 33] = 9.36, *p* = .10, Kendall’s *W* = 0.06). However, participants did not use the same decision criterion in all emotional pair conditions as reflected in response bias scores (Figure 6b). Participants showed a bias toward choosing happy faces more often than angry (*Z* = 2.73, *p* = .038) and toward choosing happy faces more than sad faces (*Z* = 2.75, *p* = .035). Fearful faces were also chosen more likely than angry faces (*Z* = 3.36, *p* = .005) in the angry–fear condition. Participants’ decision criterion was not biased when matching the neutral face in the happy–fearful pairing condition (*Z* = 0.20, *p* = 1) or in the angry–sad pairing condition (*Z* = 0.36, *p* = 1). Finally, there was no significant difference in RT for correctly matching the neutral face with the identity of one of the two emotional faces depending on the pairing condition (Figure 6c; all *p*s > 0.28).[Fig fig6]

*Summary*: In this perceptual discrimination task, we did not replicate the overall pattern of effects that we found in the VSWM task in Experiment 1 (an overall advantage for happy faces and disadvantage for sad faces). Participants’ performance in terms of overall accuracy and response times was very similar regardless of the emotion carried by the faces. This rejects the possibility that the overall VSWM results were due to happy faces being easier to match with the neutral face and to sad faces being harder to match with the neutral face. In terms of competition effects, performance (*d*′ and RT) were similar across all pairs of emotional faces suggesting no difference in discriminability between the pair of emotions that we tested. Participants showed a response bias toward selecting happy faces over angry and sad faces, and toward selecting fear faces over angry faces. These response biases in the perceptual identity matching task cannot explain the results that we found in Experiment 1 in the competition analysis (compare Figure 4a with Figure 6b). Specifically, the emotional pairs for which we found a competition effect in VSWM either did not show a difference in response bias (angry–sad and fear–happy) or showed the opposite effect (there was a bias in response to choose more happy than sad faces in the perceptual identity matching task, but we found better accuracy for sad than co-occurring happy faces in the VSWM task).

Obtaining different effects of emotion on performance in Experiments 1 and 2 is not surprising given that the VSWM task and the matching task rely on very different abilities. First, while Experiment 1 involves a spatial aspect in the task (identity relocation), Experiment 2 does not (simple identity discrimination). Second, although both experiments require some matching between the presented neutral test face and the emotional face, in Experiment 1 the test face is compared to the stored faces in memory whereas in Experiment 2 it is compared to a face that is still present on the screen. This means that in Experiment 2, participants could go through the comparison process with the actual (not remembered) face multiple times. Finally, even if it might be argued that some memory component might be involved in Experiment 2 (e.g., between fixations when comparing two faces), this would be very minimal. The three faces are presented very close to each other and can be seen simultaneously in foveal or parafoveal vision (when fixating at the centroid of all faces, they are within a radius of 4.6° of visual angle). On the other hand, in Experiment 1 the emotional faces have to be maintained in memory for 1 s before the neutral test face is presented. Thus, whereas Experiment 1 strongly relies on VSWM, Experiment 2 mainly relies on perceptual discrimination. Our results show that emotion does not affect these two tasks similarly.

Taken together, the findings of the perceptual identity-matching task indicate that the effects that we observed in Experiment 1 are unlikely to occur at a perceptual level. Instead, they suggest that the results from Experiment 1 are due to differences in VSWM for different emotional faces, thereby highlighting the role of emotional expression in VSWM for faces.

## General Discussion

The aim of this study was to improve understanding of whether and how different emotions compete for resources and, therefore, representation in VSWM. To this purpose, we presented concurrent pairs of emotional expressions during encoding that varied in valence and arousal. Participants were asked to remember the identity and location of four emotional faces, two of which shared one emotion and the other two shared a different emotion (six emotion pairs of angry, fear, happy, and sad faces). After a short delay, one of the four faces appeared with a neutral expression and participants had to report its prior location by moving the face back to its original position using a touchscreen as precisely as possible (the neutral face always shared its identity with one of the faces at encoding). To perform the task accurately, participants needed to remember both the identity and location of the four faces, while the emotion of the faces was irrelevant.

When collapsed across co-occurring emotion pairs, we found that the emotion carried by the face at encoding affected participants’ relocation accuracy overall but did not impact relocation precision. Participants were overall best at relocating the neutral probe face to its original location when it was previously seen with a happy expression, replicating the happy advantage found in this task when all faces shared the same emotion at encoding (Spotorno et al., 2018). We also observed lower VSWM accuracy for sad faces in general. When comparing VSWM within each pair of emotion, we found better accuracy for happy over fear faces, sad over happy faces, and angry over sad faces. This pattern of results is not consistent with differences in either the degree of arousal or valence between the emotional faces (as measured in Langner et al., 2010). Further, the time spent fixating the faces during the encoding period was the same independently of the emotional expression, ruling out a potential bias from a difference in encoding time. In a second experiment, we tested participants in a perceptual identity matching task. The results confirmed that the performance differences found in the VSWM task were not due to differences in the difficulty in matching the identity of a neutral face with its emotional counterpart, or to differences in response bias. We interpret our results and discuss the potential role of valence and arousal in details below. We also propose a new “alues” theory by considering the more contextualized meaning of different emotions and how this may influence memory for who was where.

### Valence and Arousal Accounts

The valence of a face modulates memory biases, with some studies showing a threat bias in visual WM (Jackson et al., 2008, 2009, 2015; Thomas et al., 2014) while others showing a prosocial happy face advantage in VSWM when identity-location binding is required (Spotorno et al., 2018; Terburg et al., 2012). The mean valence (*M*_Val_) of our stimuli was the highest for happy faces (*M*_val_ = 4.34) and around the same for the three other face expressions (*M*_val_ = 2.07, 2.03, and 1.98 for sad, fear, and angry faces, respectively). An effect of prosocial valence on VSWM for faces should, thus, predict an overall happy advantage over all other emotions. The results of Experiment 1 show that VSWM performance for relocating a neutral face was better overall if the face was initially encoded with a happy expression compared to sad and fearful faces. This general happy advantage is consistent with previous studies (Spotorno et al., 2018; Terburg et al., 2012) and indicates a prosocial advantage for remembering who was where. However, we did not find a statistically significant difference between angry and happy faces contrary to the happy versus angry benefit found by Spotorno and colleagues. This difference might be because we showed mixed emotions rather than homogenous facial expression at encoding. Indeed, when analyzing performance separately for each pair of emotion, relocation accuracy varied according to which other emotion was present during the encoding period. In this competition analysis, we found a prioritization of happy over fearful faces but not of happy over angry or sad faces. In fact, we found an opposite sad benefit over happy faces (see Figure 4a). These findings indicate that happy faces are not always prioritized in VSWM.

Arousal has also been suggested to affect WM performance such that highly arousing stimuli are better remembered (LaBar & Cabeza, 2006). In our study, happy and fearful faces had the highest average arousal, while sad faces had the lowest average arousal ratings and angry faces were intermediate. Overall, we found a disadvantage in VSWM for sad faces. This could be partially explained by an arousal account which proposes that low-intensity stimuli (sad faces in our study) are deprioritized and thus worse remembered than high-intensity stimuli (happy and fear faces in our study; Mather & Sutherland, 2011). However, when analyzing VSWM on each emotional pair basis, the degree of arousal does not account for our findings. In our experiment, we would expect the display with the largest arousal disparity, that is, for the happy and sad pairing condition, to show the highest difference in VSWM performance in favor of the highest arousing (happy) faces. Our results do show a significant difference in happy–sad paired displays, but in the opposite direction: low-arousing sad faces were better relocated. In further contradiction to an arousal-based explanation, we found (a) a happy bias in happy–fear displays despite little difference in arousal ratings between these two emotions and (b) no competition bias in fear–sad display despite a larger arousal difference between these two emotions. To sum up, the amount and the direction of any differences in arousal between paired emotions did not explain either the direction of the competition effects we found, or the absence of such effects. Therefore, arousal of the stimuli does not solely predict VSWM performance in our paradigm.

### A “SV” Account

Most studies using heterogeneous displays for investigating the competing role of emotion in WM performance have presented at encoding neutral faces together with emotional faces sharing the same emotion. Their main goal was to test how performance would change for the neutral stimuli depending on the emotional context they were seen in. Whereas one study did not find any differences in WM performance for neutral stimuli in an angry or happy context (Thomas et al., 2014), another one supports an arousal account (Lee & Cho, 2019). In their study, Lee and Cho found that when emotional (fearful or happy) and neutral faces were presented simultaneously, participants’ WM improved for the emotional faces and decreased for the neutral faces. Our study is unique as participants had to remember two pairs of emotional stimuli (using angry, fear, happy, and sad faces) presented simultaneously, and each combination of two pairs of emotions was tested. However, neither an arousal nor valence account alone can adequately support our findings.

We propose a “social value” (SV) account to try to help unify the range of effects found here and in previous research by considering how different emotional expressions evoke different interpretive responses in certain circumstances. The underlying premise is that the response to an emotion can vary depending on the context of the task and the task goals. For example, some research has shown that the value or meaning of certain emotions changes when paired with expected versus unexpected gaze direction, suggesting that expressions per se do not elicit fixed interpretations and responses. Specifically, approach emotions (angry and happy) are better recognized when paired with direct/approach than with averted/avoidant gaze, while avoid emotions (fear) are better recognized when paired with avoidant/averted than with direct/approach gaze (Adams & Kleck, 2003, 2005). Modulating effects of gaze on WM for emotional faces have also been found, wherein visual WM for happy face identities was significantly boosted when seen with averted than with direct gaze, which may suggest that the meaning behind a smile (considered sly or ambiguous with averted gaze) can be altered by other contextual features within a face (Jackson, 2018). More generally, the perceived value of items has been shown to guide how much they are prioritized in WM, both for nonface stimuli (Atkinson et al., 2021, 2022) and for neutral faces (Thomas et al., 2016). In Thomas et al.’s study, neutral faces that were imbued with a “high value” status using monetary reward were more accurately recalled from WM than faces imbued with low monetary reward. Emotional expressions can also signal different values beyond intensity or valence, such as approach (happy and angry) and avoid (fear) intentions (Adams & Kleck, 2003, 2005; Sander et al., 2007), and different emotions elicit different behavior: avoidance in response to anger, approach in response to a smile (Marsh et al., 2005; Phaf et al., 2014). In line with this, the congruent approach signal and approach behavior properties of happy faces are suggested to have perhaps elevated its value to boost VSWM in general (this study and Spotorno et al., 2018).

How could potential differences in SV account for the specific patterns of competition effects in VSWM we found here? We can only speculate, but there may be trade-offs and/or additive effects between the degree to which arousal, valence, or approach-avoid properties drive the degree of prioritization within WM in general, and within this VSWM relocation task in particular. In happy–fear pairs, where a happy advantage was found, the prosocial plus approach response behavior may have elevated the value of happy faces to boost participants’ ability to remember the location of that affiliative source. However, happy faces were deprioritized over sad or angry faces when paired with them, which could in part reflect the similarity in approach signal between happy and angry faces. Suggestive of more than an approach-avoid influence, sad faces were significantly better relocated in sad–happy pairs. Sad expressions are shown to help enhance the credibility of feelings of loss (Reed & DeScioli, 2017), so perhaps sad faces may relate to a higher empathic response and thus hold increased value over happy faces to boost WM for who was where. However, the role of empathy in attention and memory for sad faces does not appear to be well established, so this hypothesis requires further investigation. Finally, angry faces were better relocated than sad faces in angry–sad pairs, which we think could suggest that negative threat signals contain greater value for prioritization and immediate source monitoring in WM than empathy-related sad faces. Notably, fear was never prioritized relative to other expressions in VSWM, which could reflect an avoidance response especially in a relocation task where the spatial source of the original emotion signal is probed. This SV account has its limitations, as it cannot easily explain the lack of VSWM bias in either direction for happy–angry, angry–fear, and sad–fear pairs. It is likely a complex interplay of factors beyond those we have measured and tried to account for here.

### Non-WM Accounts of Our Findings

The biases in VSWM performance due to the emotion of the face at encoding might be the consequences of factors other than competition for resources in VSWM. One such factor could be the amount of attention allocated to the different facial expressions during the encoding period. Indeed, distinctive stimuli are fixated first and for a longer time, which could improve their processing (Calvo & Lang, 2005; Nummenmaa et al., 2006; Rösler et al., 2005; Thomas et al., 2014), and controlling for attentional resources at encoding can remove differences in WM performances (Lee & Cho, 2019). The analysis of eye movements in Experiment 1 (in terms of proportion of fixation time on each emotional expression) did not show any strong emotional bias during the encoding period. This shows that one emotional expression was not more distinctive and attention grabbing than another. Importantly, happy faces were not fixated for longer than angry or fearful faces when they were presented with them. This suggests that our VSWM results are unlikely to be the consequence of attentional biases during the encoding period.

A second potential explanation for the competition effects in Experiment 1 is that they were driven, at least in part, by differences in matching difficulty between the neutral face and the corresponding emotional face seen during the encoding period. Indeed, it is possible that neutral faces are structurally more similar to some emotional expressions than to others (e.g., Chen et al., 2011), thereby biasing VSWM performance for one emotion over another. However, we did not find any evidence for such effect in Experiment 2 in which no memory component was required to perform the task. Participants were as accurate and fast to match the neutral face to any of its emotional counterpart, and there was no difference in perceptual discriminability depending on the type of emotional expressions presented. Therefore, the potential difference in difficulty for matching the neutral probe face with the encoded expressive face is unlikely to have affected VSWM performance.

A third factor that might have resulted in VSWM biases is that participants would more likely relocate a face to the location of a particular emotion. The results of the perceptual identity matching task in Experiment 2 did reveal that participants’ response criterion was not uniform across emotional faces. Participants’ responses were biased away from angry faces (except when paired with sad faces), which is partially in line with previous studies showing that angry faces elicit an avoid behavioral response (Marsh et al., 2005; Nikitin & Freund, 2019; Phaf et al., 2014; Stins et al., 2011). However, VSWM performance was not lower for angry faces but even higher when paired with sad faces. Responses were also biased toward happy faces in the happy–sad and happy–angry conditions. Hence, we found both an attentional (fixation time) and a response bias toward happy faces in the happy–sad condition across experiments. However, VSWM performance was better for sad than for happy faces in this condition. Together, these results directly contradict the possibility that VSWM emotional biases could be the consequence of a response bias.

Finally, one might argue that our results could be due to low-level or identity-specific effects present in our stimulus set. We do not think it is the case for several reasons. First, all faces had similar low-level properties (contrast and luminance). The eye-movements analyses also confirm that there is no saliency bias that could explain our results. Moreover, in this study, the results are analyzed depending on the emotion of the face, pooled over all face identities. That is, the results cannot be driven by identity-specific effects: if one identity is easier to remember, this would be the case across emotions. It is possible, however, that the VSWM bias is due to an easier identification of all faces from a given emotion; but we rejected this possibility in Experiment 2. There might be item-specific effects (the combination of an identity and an emotion might render a face easier or harder to remember). To avoid those, all the stimuli were counterbalanced across participants such that all faces (identity and emotion) were presented the same number of times in different locations. Moreover, in the study by Spotorno et al. (2018) which uses the same set of stimuli, when “face stimulus” was included as a random factor in the analyses, there was no item-specific effects. Thus, we believe that our results primarily reflect the effects of emotional expression on VSWM performance.

In sum, the competition effects in VSWM that we found in Experiment 1 do not overlap with potential biases in attentional resources during the encoding period or in response criterion. Moreover, the difficulty to match the neutral face to its emotional counterparts was equal across emotions. Thus, the effects in VSWM performance in Experiment 1 are likely the results of differences in the allocation of VSWM resources depending on the emotion of the faces at encoding.

### Conclusion

In this study, we showed that when the identity and location of emotional stimuli have to be remembered and bound, VSWM is overall enhanced for happy faces (high valence and high arousal stimuli) and reduced for sad faces (low valence and low arousal stimuli). Importantly, we also found competition effects between some but not all pairs of emotions reflected by biases in VSWM for one emotion compared to the other co-occurring emotion during the encoding period. These effects were not explained by attentional, perceptual or response biases, nor by the valence or arousal of the faces. Our findings highlight the need to consider a more flexible system to define the potential role of emotions and their SV according to the requirements and the context of the task. Our proposed SV account is an attempt to move away from very specific and fixed expectations of emotion effects in memory (also common in attention and perception fields) related to high versus low arousal, positive versus negative valence, or prosocial benefits versus threat. Considering how different emotions may change in “value” (however value is defined) according to different task goals and contexts will be an important step forward in understanding emotion processing.

## Figures and Tables

**Table 1 tbl1:** VSWM Accuracy and Precision Depending on the Emotion of the Probe Face at Encoding and of the Other Co-Occurring Emotion

Test face	Angry			Fear			Happy			Sad		
Paired emotion	Fear	Happy	Sad	Angry	Happy	Sad	Angry	Fear	Sad	Angry	Fear	Happy
Accuracy (%)	63.5	68.2	70.0	67.9	65.5	65.3	68.8	73.6	65.3	61.8	61.3	71.2
*SEM*	4.2	3.3	4.5	4.0	4.3	3.8	3.7	4.0	4.0	4.4	2.6	3.9
Precision (°)	2.9	2.7	2.6	3.0	2.9	2.9	2.7	2.7	2.7	2.6	2.9	2.8
*SEM*	0.1	0.1	0.1	0.1	0.1	0.1	0.1	0.1	0.1	0.1	0.1	0.1
*Note*. VSWM = visuospatial working memory; *SEM* = standard error of the mean.												

**Table 2 tbl2:** Overall Performance in Experiment 2 Using a Perceptual Identity Matching Task Depending on the Emotion Carried by the Matching Face

Measure	Angry	Fear	Happy	Sad
Accuracy (%)	91.0	88.4	88.0	92.2
*SEM*	1.3	1.4	1.7	0.9
RT (ms)	783	788	760	773
*SEM*	21	23	21	23
BIS	0.08	−0.29	−0.11	0.32
*SEM*	0.24	0.27	0.30	0.20
*Note*. RT = reaction time; BIS = balanced integration score; *SEM* = standard error of the mean.				

## Footnotes

1 Note that we refer only to WM studies here. Tasks in which long-term memory for emotional face identities is measured tend to show happy face benefits (e.g., Chen et al., 2015; D’Argembeau & Van der Linden, 2007, 2011).

2 Linear mixed models and generalized linear mixed models were applied to analyze the data published in Spotorno et al. (2018). However, in the current study, no linear mixed models/generalized linear mixed models would allow for appropriate contrasts to test the competition effects between emotions. We thus adopted a standard statistical approach.
